# The Application of Advanced Bone Imaging Technologies in Sports Medicine

**DOI:** 10.1155/2023/7412540

**Published:** 2023-12-04

**Authors:** Samuel S. Tadros, Scott Epsley, Sameer Mehta, Brandon C. Jones, Hiran I. Rajapakse, Rashad Madi, Austin Alecxih, Daniel Kargilis, Chamith S. Rajapakse

**Affiliations:** ^1^University of Pennsylvania, Philadelphia, Pennsylvania, USA; ^2^Sports Medicine Athlete Rehabilitation & Training, LLC, Washington, DC, USA; ^3^Performance Department, Orlando Magic, Orlando, Florida, USA; ^4^Royal Medical Center, Morwell, Victoria, Australia; ^5^Quinnipiac University, North Haven, Connecticut, USA

## Abstract

Until recently, the evaluation of bone health and fracture risk through imaging has been limited to dual-energy X-ray absorptiometry (DXA) and plain radiographs, with a limited application in the athletic population. Several novel imaging technologies are now available for the clinical assessment of bone health, including bone injury risk and healing progression, with a potential for use in sports medicine. Among these imaging modalities is high-resolution peripheral quantitative computed tomography (HR-pQCT) which is a promising technology that has been developed to examine the bone microarchitecture in both cortical and trabecular bone at peripheral anatomical sites. Technologies that do not expose patients to ionizing radiation are optimal, particularly for athletes who may require frequent imaging. One such alternative is diagnostic ultrasound, which is preferable due to its low cost and lack of radiation exposure. Furthermore, ultrasound, which has not been a common imaging modality for monitoring fracture healing, has been shown to potentially demonstrate earlier signs of union compared to conventional radiographs, including callus mineralization and density at the healing site. Through the use of conventional magnetic resonance imaging (MRI), finite element analysis (FEA) can be used to simulate the structural and mechanical properties of bone. On the other hand, the ultrashort echo time (UTE) MRI can evaluate cortical bone quality by detecting water bound to the organic bone matrix and free water, providing important information about bone porosity. Several novel bone imaging techniques originally developed for osteoporosis assessment have great potential to be utilized to improve the standard of care in bone fracture risk assessment and healing in sports medicine with much greater precision and less adverse radiation exposure.

## 1. Introduction

A broad spectrum of diseases can affect bone, encompassing infectious, rheumatologic, oncologic, congenital, degenerative, and traumatic etiologies [1, 2]. Imaging provides essential information in the clinical assessment of these different types of bone diseases [2, 3]. Technologies such as dual-energy X-ray absorptiometry (DXA) have long been used to evaluate fracture risk in osteopenic patients. In addition to patients with poor structural bone integrity, athletes are another patient population who may be at an increased risk for traumatic bone injury and thus may benefit from longitudinal screening [4]. This review describes several novel imaging modalities that have been developed to assess bone health and discusses their use in sports medicine to evaluate injury risk and monitor bone healing. The technologies currently being employed as well as newer emerging techniques that may be more beneficial in the athletic population will be described. Among these techniques will be high-resolution peripheral quantitative computed tomography (HR-pQCT), which has shown promising results in analyzing bone microarchitecture at distal peripheral anatomic sites [5]. Methods such as diagnostic ultrasound, which has the ability to demonstrate earlier signs of union than conventional radiographs will be presented [6]. Both computed tomography (CT) and magnetic resonance imaging (MRI) are commonly employed to quantify trabecular and cortical bone morphology in research studies and in clinics. These imaging modalities have the additional benefit of serving to construct models of bone that can be further analyzed using finite element analysis (FEA), which will be discussed in detail [7, 8]. The emerging applications of ultrashort echo time (UTE) MRI which is currently being used to provide information about cortical bone quality will be introduced as well [9, 10].

Despite these advances in technology and the clinical utility they may provide, limitations and challenges still exist. The aim of this review is to present an overview of novel bone imaging modalities and their clinical utility in the context of sports medicine. These techniques can potentially improve the standard of care in bone fracture assessment and fracture healing in both elite and recreational athletes with greater precision and fewer adverse effects.

This review was conducted using a search of the PubMed libraries, with keywords being “sports medicine imaging,” “HR-pQCT,” “bone high-resolution MRI,” “ultrasound sports medicine,” “ultrashort echo time MRI,” “MDCT sports medicine,” and “DEXA sports medicine.” Articles were chosen based on their applicability to the stated goal of this review, namely, a clinical application in sports medicine. Other studies were included that demonstrated the utility of the imaging techniques and were deemed by the authors to be critical “technological advancement” or “proof of concept” papers. The excluded studies were those without a clear clinical correlation or focused strictly on technical aspects of the imaging modalities. This review is not meant to be completely extensive. Rather, it is structured to introduce readers who may not routinely stay up-to-date with radiology or sports medicine literature to the emerging technical developments that are taking place in the field.

## 2. Need in Sports Medicine

The prevalence of bone injury in athletes is elevated compared to the general population. For example, it is estimated that up to 40% of athletes will experience a stress fracture at some point in their career [4]. These injuries are painful, impair performance, and can result in permanent disabilities, financial burdens, and a loss of playing time. Stress fractures account for around 10% of all orthopedic injuries and about 20% of injuries seen in sports medicine clinics, highlighting the healthcare burden of these injuries [4]. Most of these stress fractures (80–95%) occur in the lower extremities [4]. Professional athletes, particularly basketball players, subject their lower extremities to significant repetitive loading during both regular-season and off-season training. Khan et al. identified all bony stress injuries from 2005 to 2015 in the National Basketball Association (NBA) and found that 55% of lower extremity stress fractures involved the foot, with over 80% of those injuries occurring during the regular season and a half of those occurring within the first 6 weeks of regular season action [11]. The most concerning of these injuries are stress fractures to the fifth metatarsal, with over 40% of athletes who sustain this injury are unable to return to professional play [11]. However, these injuries do not exclusively affect those playing at the professional level. Ruddick et al. studied over 11,942 injuries sustained by Australian high school athletes over a three-year period [12]. They found that stress fractures in the foot and lumbar spine were the most common, accounting for 30% and 23% of all reported stress fractures, respectively. Gymnasts had the highest frequency of lumbar spine stress fractures due to the nature of the sport, whereas rowing athletes had the highest frequency of rib stress fractures. In addition, female athletes were more likely than their male counterparts to have a stress fracture [12]. Across all sports, the foot was the most common location for stress fractures. This data suggests that it is critical for sports medicine providers to screen for these injuries and assess bone health to determine the risk of injury.  Table 1 compares the technical capabilities of imaging bones of the extremities, namely, the radius and tibia, to the deeper structures of the femur and vertebrae.

## 3. Dual-Energy X-Ray Absorptiometry (DXA)

Before delving into emerging imaging technologies, it is essential to discuss the existing techniques used to assess bone health. Medical professionals who routinely assess for osteopenia in their patient populations are familiar with the dual-energy X-ray absorptiometry (DXA) scan. This radiographic method is used to determine a patient's bone mineral density (BMD) and is the current gold standard. DXA relies on the principle that bone and soft tissue attenuate X-rays differently. As a single X-ray beam passes through the body, it is attenuated by both bone and soft tissue, making it impossible to determine the bone's contribution to the attenuation. However, the attenuation coefficient ratios of bone to soft tissue vary with the X-ray energy. By using two different energies of X-ray, one can apply the difference in total absorption to isolate the attenuation contribution from soft tissue. The soft tissue contribution is then removed, leaving just the contribution of bone [13]. The received radiation energy per pixel is quantified and converted into an “areal density,” measured in g/cm^2^. A summation of the pixels within a specified area facilitates the calculation of BMD. These values can be represented in units of g/cm^2^ or transformed into normalized *T*- and *Z*-scores [13]. A *T*-score compares the patient's BMD to that of a young adult's peak BMD. According to World Health Organization (WHO) guidelines, bone health is classified based on *T*-scores: a *T*-score of −1.0 or above indicates normal bone density, between −1.0 and −2.5 suggest osteopenia, and a score of −2.5 or lower confirms osteoporosis.

In addition, *Z*-scores provide a comparison of a patient's BMD with that of age- and weight-matched peers [14]. However, technical challenges remain that complicate the use of DXA scans in sports medicine, such as the fact that BMD is derived from two-dimensional DXA images, which lack microstructural information about bone architecture, thus, rendering them inaccurate in predicting bone strength and fracture risk in athletes [15]. Furthermore, DXA measurements are typically performed in the hip or lumbar spine, while most sports-related bone injuries occur in the peripheral skeleton. It has been shown that changes in bone quality in the peripheral skeleton are only weakly or moderately correlated with measurements obtained from the axial skeleton [16]. Although radiation is involved, the amount is minimal and much less than that of a standard chest or dental X-ray. Such results have spurred interest in finding better ways to evaluate bone strength and fracture risk beyond regular metrics like BMD for more practical use in a sports medicine population.

On the other hand, using DXA to measure body composition in athletes has been gaining popularity, mainly by harnessing existing technological capabilities. Previous work by Mazess et al. established DXA's accuracy in assessing body composition, demonstrating that DXA results closely aligned with reference materials of lard and water, representing fat and lean tissues, respectively [17]. Numerous studies have conducted comparative analyses between DXA and other existing body composition measurement techniques. The findings indicate no significant differences, thereby establishing DXA as a reliable instrument for body composition assessment [18, 19]. A comprehensive DXA protocol for young athletes often encompasses assessments of the hip, spine, and total body to evaluate osteopenia, low adiposity, and insufficient muscle mass. DXA's growing popularity in sports medicine is primarily attributed to its body composition measurement capabilities [20].

## 4. Computed Tomography (CT)

Since its introduction in the 1970s, computed tomography (CT) has dramatically changed medical imaging and has enhanced diagnostic imaging capabilities for a myriad of diseases. CT enables the detection of bone disease and enhanced visualization of complex fractures, especially peri-articular and intraarticular fractures which may need to be visualized in several different planes for appropriate surgical planning. The CT scan utilizes rotating X-ray beams to generate cross-sectional images of targeted body regions. As the X-ray beams traverse the body, they are attenuated and captured by detectors. Then, the collected data is algorithmically processed to produce individual CT slices [21]. Advances in CT technology have enabled higher scan resolution, lower ionizing radiation, and shorter scan times, allowing for more complex and precise image analyses that can be used to quantify bone health. In addition to the standard clinical CT machines, there are two specific types of specialized CT machines. These are high-resolution peripheral quantitative computed tomography (HR-pQCT) and multidetector computed tomography (MDCT), both of which are especially useful for sports medicine and are detailed below.

### 4.1. HR-pQCT

High-resolution peripheral quantitative computed tomography (HR-pQCT) is a CT variant that is a noninvasive, low-radiation modality for evaluating bone microarchitecture and volumetric BMD in both cortical and trabecular compartments [5]. Second-generation HR-pQCT devices have the ability to achieve an isotropic voxel size of 62 *μ*m, which allows direct microstructural imaging of the bone. Similar to a conventional CT scan, this imaging modality uses computerized processing of X-ray attenuation, measured in Hounsfield units, to acquire sectional images, which are then processed to reconstruct a 3D model of the bone. The distal radius and tibia are the most common sites imaged with HR-pQCT since the scanner's gantry is relatively narrow and shallow; hence, it can only accommodate the peripheral skeleton. The patient's extremities are typically immobilized in a carbon fiber shell to reduce motion artifacts that can arise during image acquisition [22].

This technology has been commercially available since the mid-2000s but its use in the clinical research space has dramatically increased over the last decade. HR-pQCT has been used to quantify age-related changes and sex differences in bone microarchitecture, the effects of bone metabolic disorders, and the response of bone to various osteoporosis therapies. Higher intracortical pore volume indicates impaired bone remodeling and diminished bone strength [21]. Furthermore, more porous bone has been shown to have weaker biomechanical properties [23].

**Figure 1:**
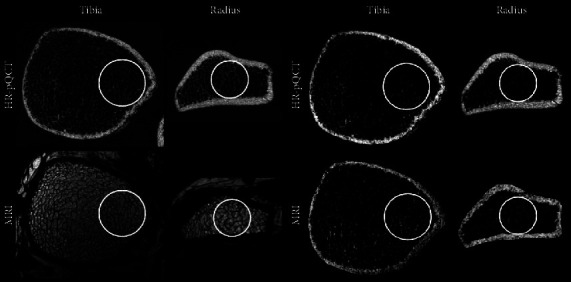
Comparison of bone microstructural images in the same subject between HR-pQCT (top) and high-resolution MRI (bottom) in the distal tibia and radius. The HR-pQCT scan was taken at 82 *μ*m isotropic voxel size using standard protocols. The high-resolution MRI scan was performed using a previously described protocol with in-plane resolution of 137 *μ*m [16, 24]. The images to the left display the raw images taken which include the background soft tissue and the images to the right show the images segmented and the MRI scan processed such that the signal intensity is inverted. It is clear that, although the modalities have different contrast, both produce similar information about bone microarchitecture.

HR-pQCT has often been studied in conjunction with more widely available and better-validated technologies such as MRI. Image acquisition between HR-pQCT and high-resolution MRI demonstrates strikingly similar qualitative results (Figure 1). Many studies have also validated the fact that both methods produce similar quantitative results. Using both *in vivo* and *ex vivo* models, high correlations between the two techniques were found for trabecular number and spacing, both of which are common quantitative measures of trabecular bone health. Furthermore, it was found that both HR-pQCT and MRI correlated well with gold standard µCT values obtained *ex vivo* [25]. Accounting for these discrepancies is critical for sound interpretation, and one should not compare absolute values between modalities but instead restrict comparisons to within each modality. High-resolution MRI techniques will be discussed later in this review in greater detail.

The compelling potential of HR-pQCT technology lies in its ability to assess and predict fracture risk, and it is this application that is particularly powerful and relevant to the future of sports medicine. Steven Boyd's group at the University of Calgary used this technology to evaluate 95 athletes aged between 16 and 30 across an array of sports [26]. Using HR-pQCT-based bone parameters including total BMD, trabecular BMD, number, thickness, and separation, as well as cortical thickness and porosity, they were able to validate existing data showing that impact loading is positively associated with bone quality. Expanding on this, it has been shown that bone microarchitecture biomarkers in elite athletes participating in impact-loading sports were elevated, indicating increased bone strength [26]. The same group at the University of Calgary used HR-pQCT to assess the subchondral bone microarchitecture of ACL-reconstructed knees in young female athletes through comparisons with their uninjured contralateral knee and with healthy aged-matched female control knees with no injury history. The investigators found that the ACL-reconstructed knees had reduced trabecular BMD and a thickened subchondral bone plate in the lateral femur, both of which are signs of early-stage osteoarthritis [27]. HR-pQCT can also be used to assess the efficiency of rehabilitation following injury. In a published case report, HR-pQCT was used to evaluate the bone quality of an individual with a complete motor deficit due to a spinal cord injury whose disuse osteopenia was treated by functional electric stimulation- (FES-) rowing training. It was found that the subject had bone microarchitecture and density similar to noninjured age-matched controls, suggesting diminished bone loss compared to chronic spinal cord injury patients without FES training [28]. These results highlight the utility of HR-pQCT in clinical sports medicine and rehabilitation, as well as the versatility of this technology in clinical research.

Despite this technology's promise, certain limitations warrant judicious clinical application. HR-pQCT has been shown to be a reliable method for evaluating changes in bone strength, but future studies are needed to determine its usefulness fully [29]. Furthermore, HR-pQCT and its associated image analysis methods and software were designed and validated for mature bone, but it is yet to be validated on immature bone [30]. By the same token, evidence is still emerging on whether the distal radius or tibia is the ideal site for monitoring therapy directed toward osteoporotic patients susceptible to lumbar spine or proximal femur fractures [5]. Moreover, the segmentation process of HR-pQCT has challenges associated with differentiating cortical from trabecular bone as the precise border; it is not always present due to actual biological effects in the endocortical and intracortical envelopes [5]. Also, some other limitations center around technical issues related to HR-pQCT. Partial volume effects, beam hardening, reconstruction challenges, and inherent assumptions in calculating all the structural and densitometric measures should be interpreted judiciously [5]. In particular, beam hardening is a common artifact in HR-pQCT resulting from overattenuating lower-energy photons. While this can be attributed to numerous phenomena, in practice, it is most commonly a result of large body habitus or extreme variation in each subject's body composition [31].

Another notable limitation is the technology's limited accessibility worldwide since it is currently still a research tool and has yet to be adopted in standard clinical practice. For example, in 2013, there were only 45 HR-pQCT systems worldwide, compared to tens of thousands of DXA, CT, and MRI machines [5]. Before being adopted for common place use in clinical practice, further evidence to show the utility of HR-pQCT in fracture prediction is necessary either in conjunction with DXA or in lieu of DXA. In the sports medicine setting, particularly at the professional level, organizations are searching for valid and reliable data to compare to the same cohort to gain a competitive advantage in their respective leagues. Thus, the development of normative data will provide a better context of outcome measures from HR-pQCT, similar in concept to how *T*-scores are routine metrics written on DXA scan reports [5].

### 4.2. Multidetector-Row Computed Tomography (MDCT)

**Figure 2:**
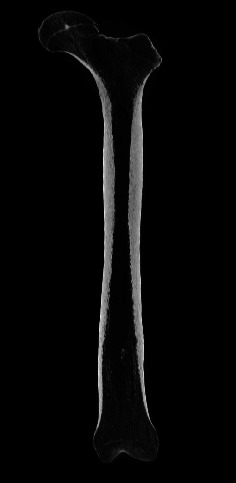
A coronal slice of a MDCT scan of a fresh, frozen cadaveric femoral specimen. The MDCT protocol has an in-plane resolution 400 *μ*m. It is clear that MDCT provides excellent contrast and direct imaging of trabecular microstructure.

Multidetector-row computed tomography (MDCT) was first introduced in 1998 and has seen remarkable growth in terms of applicability and usage over the last two decades [32]. Single-detector CT scanners utilize a single row of detector elements that form an arc in the axial plane and allow for the acquisition of a single slice image from one X-ray tube rotation. In contrast to these previous technologies, MDCT scanners leverage multiple detector rows in the *z*-axis, which creates a two-dimensional curved detector array. Modern MDCT scanners utilize 64 or more rows of detector elements. This allows MDCT scanners to acquire more than one slice with a single X-ray tube rotation, resulting in submillimetric slices and high-resolution images of the trabecular bone microstructure (Figure 2). MDCT scanners offer several advantages over older technologies, such as faster scanning times, higher resolutions, broader *z*-axis coverage, and isotropic scanning. MDCT scanners can also provide 3D bone microstructural information and improve bone health and fracture risk measurement [3, 29, 33, 34]. This has expanded its use in the assessment of a variety of pathologies, including those encountered in the field of sports medicine.

Despite the promise of this technology, there are certain challenges associated with its use. Most noteworthy among them is the radiation exposure related to scanning. The bulk of the research surrounding MDCT bone imaging is focused on determining whether it is possible to reduce radiation exposure to an acceptable safety profile while maintaining adequate image quality and clinical utility. This is particularly important in bone evaluation since frequent scanning may be required to detect longitudinal changes from extensive training regimens or recovery following injury; hence, radiation accumulation remains a concern.

Low-radiation MDCT scans would also likely be useful for sports medicine applications. MDCT arthrography has been used to evaluate the severity of cartilage damage and scapholunate dissociation, finding that the chondral damage is more severe when the scapholunate interosseous ligament is completely ruptured [35]. Another group published a case series of 11 patients who underwent unilateral double-bundled anterior cruciate ligament reconstruction with hamstring tendon autografts. They evaluated volume changes in the bone tunnel using MDCT following surgery and found that at one year, the bone tunnel had enlarged slightly though this finding was not statistically significant [36]. Other orthopedic surgeons have successfully used MDCT to evaluate the bone bridge between the bone tunnels following anatomic double-tunnel ACL reconstruction [37]. Due to the highly accurate spatial volume resolution offered by MDCT, researchers have applied it to study the segmental rotation of vertebrae in the lumbar spine as well as the movement of facet joints [38]. These comprise exciting clinical applications of MDCT technology but only scratch the surface of their ultimate potential utility in the world of sports medicine.

## 5. Finite Element Analysis (FEA)

As promising as these modalities are for bone diagnostics individually, the true potential of HR-pQCT and MDCT is in their ability to be used in conjunction with FEA to compute bone strength directly. While bone density metrics obtained from DXA are a reasonable proxy of true bone strength, many fractures occur in individuals with bone mineral densities that are in the normal range [5]. FEA is an established computational framework that has been used in engineering and mathematics for some time and is now showing promising results in assessing bone health. Essentially, FEA is a computational method to solve partial differential equations similar to those seen in structural analyses, heat transfer, mass transport, electromagnetic attractions, and fluid flow [39, 40]. In the context of bone, solid mechanics predict how a given material will respond to an applied force or stress. It works by taking a generated mesh, which in this context is a three-dimensional bone model obtained from imaging, and dividing this input into numerous smaller elements, namely, voxels. By assuming the material properties of bone, such as the elastic modulus, which has been shown to be related to voxel-specific image intensities [41], the model can simulate how each of these constitutive elements would respond to a simulated loading condition, thereby enabling computation of the bulk biomechanical properties of bone, such as yield strength, ultimate strength, and stiffness to name a few [7, 42]. These kinds of studies can be performed *ex vivo*, as there is evidence that formalin fixation and freezing do not significantly impact trabecular bone microstructure or finite element modeling, and such *ex vivo* modeling experiments have contributed a great deal to the literature in this area as well as defining the clinical utility of this technology [43]. Given the ability of HR-pQCT and MDCT to generate accurate three-dimensional models of bone microstructure that could be applied as a mesh for FEA, several groups have been interested in using FEA to calculate bone strength and predict fracture risk in athletes. Although several studies have developed patient-specific finite element models using segmented BMD maps from DXA scans, these models are limited by the 2D representation of DXA images, which do not capture the irregular three-dimensional geometry and spatial distribution of bone and the variations in cortical and trabecular bone in humans [44–46]. These limitations can result in discrepancies in the predicted loading patterns compared to actual 3D modeling in CT and MRI, consequently limiting the adoption of DXA-based finite element modeling in the field of sports imaging.

Finite element analysis evaluation of bone strength has shown immense promise in clinical and sports science applications. Schipilow et al. studied the effect of impact-loading sports on bone microstructure with HR-pQCT FEA and found that FEA-predicted failure load was associated with higher-impact sports and greater muscle strength [26]. Anitha et al. also utilized FEA to compute failure load with MDCT data analyzing vertebrae with and without fracture [47]. It has also been shown that FEA is still feasible even in the context of radiation dose reduction with MCDT imaging, as dose reductions of up to 64% did not impact finite element strength estimates [47]. Researchers have utilized an FEA model to evaluate the effect of an aggravating medial meniscus tear on knee osteoarthritis by using metrics like peak shear stress, peak compression stress, and meniscus extrusion calculated from finite element simulations [48]. They were also able to apply FEA to predict the implications of the resultant meniscectomy from those knees with aggravating meniscus tears. They found that meniscectomy also increased the stress calculations from the model, likely due to an increase in the meniscus extrusion metric [49]. Using this data, the authors concluded in a subsequent paper that longitudinal tears to meniscal horns increased the magnitude and changed the stress distribution in the knee, likely leading to subchondral bone osteonecrosis [49]. Another group used finite element simulation to study the loading forces on the human shoulder, finding that maximum stresses occurred in the lower half of the surrounding cartilage and that compression tensions increase as humerus-scapula contact increases [50]. These are all exciting examples of the utility of FEA in sports medicine. Its utility will likely grow in assessing athletes' fracture risk and monitoring articular stress pre-and post-operatively.

## 6. Ultrasound

Diagnostic ultrasound has become an increasingly popular tool in the point-of-care evaluation of bone injury, primarily due to its cost-effectiveness, accessibility, multiplanar imaging capability, and lack of ionizing radiation [6, 51]. Furthermore, ultrasonography of bone injuries has been proven effective beyond simply fracture diagnosis, with utility described in the monitoring of bone healing. Its use has now been expanded to the sideline evaluation of bone injuries where radiographs are unavailable, early diagnosis of stress fractures, detection of occult fractures, and monitoring of the reparative process [6, 52–54].

The diagnosis of bone injuries with ultrasound can be made using an algorithm that includes clinical suspicion, local swelling, palpable tenderness, and a number of sonographic criteria. Evaluation is performed in at least two planes using B-mode ultrasound and a linear array transducer with a frequency range between 5 and 17 MHz [55–57]. Fractures are clearly identified as a defect in the bright hyperechoic cortex accompanied by periosteal elevation, local soft tissue edema, and a hematoma. Features of stress fractures include a dark hypoechoic periosteal elevation at the site of tenderness and hyperemia when evaluated with a power Doppler [6, 51, 52]. The accuracy of ultrasound compared to conventional radiography in diagnosing fractures is as high as 100% at several anatomical sites, including the ribs, sternum, and femur [55]. Sensitivity and specificity for long bone fractures were reported as 90.2% and 96.1%, respectively, by Waterbrook and colleagues [56]. As for stress fractures, Banal et al. [51, 52] reported a detection sensitivity and specificity of 83.3% and 75.9%, respectively, noting associated rheumatologic disease as a confounding factor.

Although ultrasound can be a very useful adjunct in imaging fractures and stress fractures, it is not without its limitations. Accuracy decreases when imaging the proximal or distal ends of long bones [56]. In particular, distal, intraarticular bone injuries may appear hypervascular on Doppler and may be diagnosed as synovitis, missing the fracture, partly due to the irregular contour of the bone in those regions [51]. Furthermore, ultrasound is unable to image intraosseous pathology. Bone irregularities such as nutrient vessels, physeal plates, shadowing from sesamoids or ossicles, and cortical erosion have been cited as frequent causes of misdiagnoses [6].

**Figure 3:**
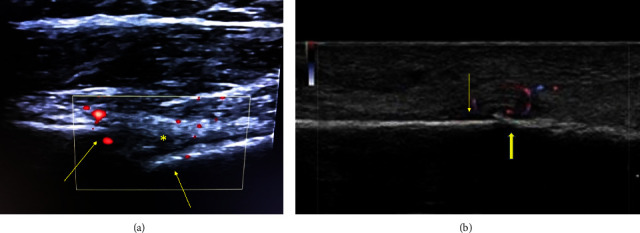
(a) Long-axis sonogram of the proximal humerus demonstrating fracture. Note the interruption of the hyperechoic cortical line (arrows). An echogenic hematoma (asterisk) is identified in the fracture space, and early vascularity is detected on power Doppler (red). (b) Long-axis sonogram of a fibular stress fracture. Periosteal elevation (arrow) is identified, along with increased Doppler activity (color) and subtle cortical irregularity at the site of pain (solid arrow).

An emerging application of diagnostic ultrasound in sports medicine is the monitoring of fracture healing. Wright et al. were one of the first to discuss this application, suggesting that ultrasound may be useful in monitoring fracture healing following intramedullary screw fixation of a Jone's fracture of the fifth metatarsal [53]. They further posit that while imaging alone cannot determine a safe timeframe for return to sport, it is helpful to understand the healing status to make a better decision. The sonographic appearance of the three phases of healing: reactive, reparative, and remodeling, has been described [54]. A hematoma adjacent to the fracture site 2-3 days postinjury was observed during the reactive phase, followed by increased vascularity between weeks 2 and 4 in the reparative phase (Figure 3(a)). Early calcification, seen as small hyperechoic specks, could also be visualized in this phase, indicating early fracture healing. In contrast, plain radiographs were only able to demonstrate changes associated with the late reparative and remodeling phases while failing to identify early calcification. Complications at each phase, including delayed union, nonunion, fibrous union, and infection, may also be best detected early through the use of ultrasound [54, 58].

Furthermore, Doppler flow has also been applied towards identifying phases of fracture healing and shows a distinct pattern compared to delayed union. In Doppler ultrasound, the frequency of the reflected ultrasonic wave varies depending on the object's motion relative to the transmitter. This variation in frequency, known as the Doppler effect, allows for noninvasive measurement of blood velocity and subsequent calculation of flow within the body [59]. When applied to fractures, serial ultrasound scans demonstrated no Doppler flow immediately postsurgery in patients with tibial fractures that were treated by external fixators. On the other hand, serial ultrasounds demonstrated an increase in flow and a decrease in resistance index associated with greater vessel density in healing fractures (Figure 3(b)). Fractures that resulted in delayed union did not demonstrate an early increase in Doppler flow and, conversely, were characterized by an increase in resistance index over time due to poor vessel density upon identification of vascularity [60].

**Figure 4:**
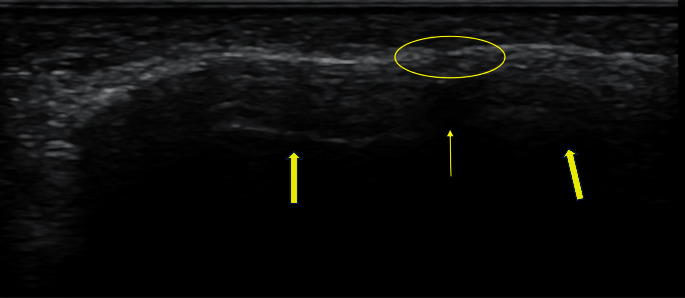
Long axis image of a healed fracture (circle). The cortical line can be seen to have been restored. Reverberation artifact seen deep to the cortex and adjacent to the fracture site (solid arrows) is replaced by acoustic shadowing (arrow) suggesting decreased mineralization of bone at the fracture site.

A less well-recognized feature of fracture healing on ultrasound evaluation is acoustic shadowing. The normal bone cortex is highly echogenic at its surface, demonstrating a reverberation artifact deep to the cortical line [6]. In monitoring the healing of tibial tuberosity osteotomies in dogs through the use of three imaging modalities (US, CT, and X-ray), Risselada and colleagues were one of the first to identify acoustic shadowing as a key feature of bone healing [57]. They defined healing on ultrasound as a hyperechoic line at the previous osteotomy gap, with acoustic shadowing indicating mineralization (Figure 4). In this study, cortical bridging was visualized on ultrasound between 1 and 2 months, and acoustic shadowing by 3 months. They further noted that ultrasound was able to diagnose fracture healing sooner than both plain radiographs and CT scans. This can be helpful when serial monitoring of fracture healing is desired without repeated exposure to ionizing radiation.

In summary, ultrasound is a cost-effective, safe, and sensitive alternative to diagnosing fractures and stress fractures and monitoring bone healing. Ultrasound is, however, limited in its ability to observe intraosseous pathology and has decreased sensitivity at the proximal and distal ends of long bones, and as such, awareness of concomitant pathologies or anatomic variants that can be misleading is critical. The ability to serially image and track healing over time has the potential to impact critical clinical decisions such as when to increase the bone load and the early identification of malunions. This may prove to be one of the ultrasound's greatest advantages over other imaging modalities.

## 7. Magnetic Resonance Imaging

**Figure 5:**
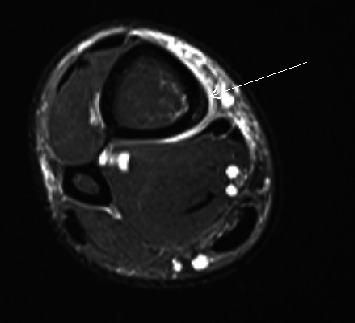
Clinical axial T2 fat sat image in a 40-year-old male demonstrates intracortical and periosteal edema (arrow), consistent with medial tibial stress syndrome.

Magnetic resonance imaging (MRI) was developed in the 1970s and 1980s, and since that time, it has been used for a variety of medical applications. Similar to ultrasounds, MRI is preferable in that it does not expose patients to ionizing radiation and, therefore, is safe for repeat scans. Although it is more expensive and requires a longer scan time than other imaging modalities, MRI is widely available and can assess the whole body, and many clinicians are familiar with this technology due to its proven track record in diagnostic imaging and biomedical research. In contrast to X-ray-based techniques, wherein each tissue has a prior known density with bone being the most hyperintense, the signal and contrast that results from MRI scans are highly tunable. The basic principle of MRI is that, in the presence of a large homogenous magnetic field, certain atomic nuclei can be perturbed by an orthogonal radiofrequency (RF) magnetic field pulse. This perturbation creates an RF signal that is detected by the radiofrequency coil within the scanner. Since the resultant signals are highly dependent on the timing of the RF pulses and readout, these can be manipulated to detect primarily fat (called a T1-weighted scan) or fat and water (called a T2-weighted scan), as well as numerous other combinations of tissues. Indeed, the highly tunable nature of MRIs makes them suitable for the clinical assessment of various musculoskeletal tissues, including bone, tendons, ligament, cartilage, and fibrocartilage [61]. For example, medial tibial stress syndrome, a condition commonly known as “shin splints,” is caused by intracortical and periosteal edema, which can be visualized on T2-weighted scans (Figure 5) [62, 63]. In addition, chemical shift-based MRI imaging is especially useful for imaging fatty infiltration of the rotator cuff and is associated with rotator cuff tear severity, age, and isometric muscle strength [64]. Furthermore, MRI quantification of T2 relaxation times is capable of measuring cartilage health and is linked to osteoarthritis progression due to aging and athletic injuries [65, 66]. These are just a few of the multitudinous MRI applications for the musculoskeletal system; an exhaustive overview is beyond the scope of this review. We instead will focus on the development, clinical research, and potential sports science applications of MRI in bone diseases. For an extensive overview of MRI's role in evaluating the entire musculoskeletal system, readers may refer to the work of de Mello et al. [61].

There are numerous methods for MRI evaluation of bone health that are used clinically, both through direct imaging of bone marrow and cartilage as well as through indirect imaging of trabecular and cortical bone. It is worth pointing out that trabecular bone and cortical bone have different biomechanical properties and varying physiological roles in the body and this certainly impacts imaging considerations as well as therapeutic approaches [7]. Trabecular bone is typically found in the spine, hip, and wrist and is subject to consistent bone remodeling and consequently increased fracture risk. Compared to trabecular bone, cortical bone is much more compact and found primarily in the shafts of long bones such as the femur and tibia, comprising approximately 80% of the skeleton. Cortical thinning and increased porosity are important predictors of fracture risk and bone strength [67–69].

MRI finite element modeling techniques like those described earlier can be applied to high-resolution MRI scans of bone microstructure to directly compute biomechanical properties such as stiffness, elastic modulus, and failure load [8, 70, 71]. It has been demonstrated that elastic moduli computed from simulated MRI models were highly correlated with those obtained from CT-derived models, alleviating concerns that noise and lower resolutions from MRI would diminish the efficacy of FEA [72].

**Figure 6:**
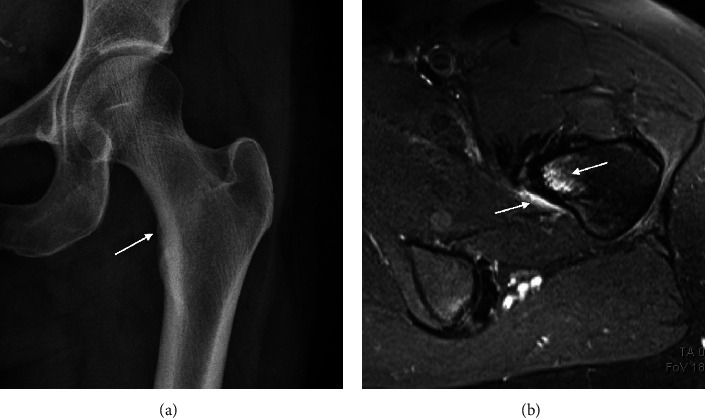
(a) X-ray of a 37-year-old female showing cortical thickening (arrow) along the medial femoral neck inferiorly in keeping with a stress reaction. (b) Axial T2 FS MRI showing marrow edema and periosteal edema, confirming stress reaction.

The extensive use of MRI in the clinical assessment of bone and cartilage is indicative of the modality's usefulness in the field of sports medicine, and several groups have utilized this technology to address indefinite research questions in sports medicine. One group studied the effect of high-impact exercise on femoral neck bone mass, a common site of fracture in osteoporotic patients and a leading cause of morbidity. They looked at 42 postmenopausal women and had them participate in 6 months of progressive high-impact exercise on one leg then compared the bone mass in this leg to the contralateral unexercised leg using both DXA and MRI of the knee joint. They found that in the exercised leg, there was an increase in BMD, bone mineral content, and section modulus at the femoral neck, while MRI analysis showed no difference in articular cartilage quality between the exercised knee and the contralateral unexercised knee. Bone marrow lesions and cartilage defects were present, particularly in the patellofemoral joint, although there was no evidence that the intervention led to a progression of osteoarthritis [73]. This data suggests that high-impact exercise is a reasonable intervention to strengthen the femoral neck and potentially reduce osteoporotic fractures. This strengthening can be visualized on X-rays (Figure 6(a)) and marrow edema consistent with the stress reaction can be visualized on T2-weighted MRI (Figure 6(b)). Another study was interested in the impact of physical activity on the proximal humerus, an underappreciated site of osteoporotic fracture. They answered this question by comparing the throwing arm to the nonthrowing arm in professional baseball players. With high-resolution MRI, they looked at the humeral head and greater tuberosity and found that adaption of the trabecular bone at the proximal diaphysis was occurring, especially in the posterolateral cortex in a pattern that approximated the strain distribution in the shoulder joint during a pitch [74]. This not only suggests that physical activity can strengthen bone in a clinically relevant site but also further elucidates the relationship between mechanical loading and skeletal adaptation. MRI has also been used extensively to study the biomechanical forces on the knee. One set of investigators wanted to compare two methods of femoral tunnel drilling in ACL reconstruction, the mini-two incision method and the anteromedial portal technique, regarding knee kinematics and early cartilage degeneration. Twenty patients were evaluated with MRI one year after their ACL reconstruction, and quantitative cartilage imaging was performed and compared to control knees. It was found that while both techniques allow for an anatomic reconstruction, knees repaired with the mini-two-incision method showed more early cartilage degeneration [75]. Another group used MRI to study longitudinal changes in subchondral bone 18 months following a high tibial osteotomy. They found that trabecular bone thickness increased in both the lateral femur and tibia and was associated with better functional outcomes [76]. However, MRI technology is not limited to the assessment of surgical outcomes. MRI-derived finite element modeling was used to study the biomechanical effects of a valgus unloader brace in the knee following a medial meniscectomy. It was found that increasing valgus alignment resulted in a reduced contact force in the medial compartment, but increased contact in the lateral compartment could potentially predispose the patient to early-onset osteoarthritis [77].

## 8. High-Resolution MRI

**Figure 7:**
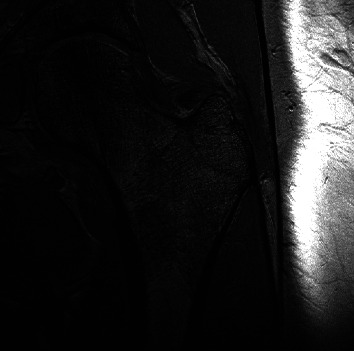
A coronal slice of a high-resolution MRI scan of the proximal femur demonstrating the ability of MRI to image trabecular bone microarchitecture in deep sites. The high-resolution MRI scan was obtained with a spoiled gradient recalled acquisition in steady state sequence with an in-plane resolution of 234 *μ*m using previously described protocols [88].

Several of the studies that have been discussed employed high-resolution MRI imaging. It is important to highlight this technology in greater detail, as it represents a significant advancement in the field and lends itself well to sports medicine. Historically, high-resolution MRI analysis of deep bone structures such as the proximal femur was limited by poor signal-to-noise ratios; however, improvements in hardware and the advent of optimized pulse sequences made it possible to reliably depict and quantify the trabecular microarchitecture in the proximal femur [78]. Technical standards are described in the literature (Figure 7) [79, 80].

It has been shown that there are strong correlations (*R* = 0.86) in structural parameters between DXA and 3 Tesla MRI at the femoral head and trochanteric region [78]. This study demonstrated the feasibility of high-resolution MRI, but further technological advancements have been made. Among these is the use of a variable flip angle three-dimensional fast spin-echo (3D VFA-FSE) sequence combined with outer volume suppression to image the trabecular bone structure at the proximal femur. This 3D VSA-FSE sequence was compared with a previously demonstrated multiple-acquisition 3D balanced steady-state free precision (bSSFP) using a theoretical simulation and found to have at least a 35% higher signal-to-noise ratio and minimal blurring. These results were validated with *ex vivo* and *in vivo* studies [81]. Furthermore, since the accuracy of bone FEA models is dependent upon resolution [82–85], these high-resolution MRI images are especially useful when used in conjunction with bone FEA and provide the most accurate assessments of bone strength possible. High-resolution bone MRI FEA has demonstrated a high degree of *in vivo* reproducibility in the literature [86, 87]. Moreover, the accuracy of the hip strength estimates as calculated by FEA has been validated with cadaveric studies. Researchers have directly tested cadaveric human femurs using the same loading conditions simulated in their finite element models. They observed strong correlations between the biomechanical properties of the hips as measured directly versus those predicted by their simulations, suggesting that their model could accurately predict bone strength in a clinical setting [85].

The use of high-resolution MRI is not limited to the proximal femur. Warden et al. used it to assess the shoulder joint as was discussed earlier [74]. High-resolution MRI has also been used to study the distal femur. Chang and colleagues were interested in comparing bone mechanical stiffness in the distal femur of competitive female dancers compared to healthy, relatively inactive female controls matched for age, weight, and height. They recruited 9 modern female dancers and 10 controls and scanned their distal femurs with a 7 Tesla scanner at high resolution, and subsequently used FEA to compute bone stiffness, cross-sectional area of whole, cortical, and cancellous bone, as well as cortical thickness. They found that the dancers had greater whole and cancellous bone stiffness, although cortical bone stiffness did not differ [89]. This suggested that the elevated whole bone stiffness in dancers may be mediated via cancellous rather than cortical bone adaption. The use of high-resolution MRI in studies such as these will only accelerate in the coming years as this technology becomes more accessible. Furthermore, since high-resolution bone MRI FEA has a high degree of accuracy and reproducibility for assessing bone strength and does not produce ionizing radiation, this method is uniquely suited for longitudinal studies evaluating changes in bone strength over time. One particular group that could benefit from this is professional sports teams who want to track bone remodeling either during intense training or during recovery from injury to gain a competitive advantage.

## 9. Ultrashort Echo Time (UTE) MRI

As we have discussed, MRI can be used to reconstruct trabecular bone marrow networks for further analysis, but imaging of cortical bone is more complicated as it is much denser than trabecular bone. Under typical conditions, the cortical bone signal is close to background intensity and is essentially undetectable with conventional MRI. About 20–30% of cortical bone volume is composed of water, but it cannot be acquired by conventional MRI because of its short T2 transverse relaxation time [8]. In the last decade, the development of ultrashort echo time (UTE) MRI made the detection and quantification of this water possible *in vivo* [23, 90]. Comparison with *ex vivo* specimens of the tibial mid-shaft showed that UTE MRI was able to quantify bulk bone water accurately [91]. The majority of this bone water resides in the Haversian and lacunar-canalicular systems that permeate cortical bone and is therefore associated with cortical bone porosity. This metric is clinically relevant as it is known to increase with age and in osteoporosis as well [67, 91]. Moreover, a cadaveric study of 235 cortical bone specimens of the proximal femur found that increased porosity was associated with a decline in mechanical properties such as ultimate stress, ultimate strain, and energy absorption [92]. The ability of UTE MRI to detect tightly bound water has enabled direct assessment of collagenated connective tissues like articular cartilage, tendon, ligaments, and bone visible on imaging [9, 93], proving its clinical utility. These measurements could then be used to further improve FEA simulations of bone strength.

**Figure 8:**
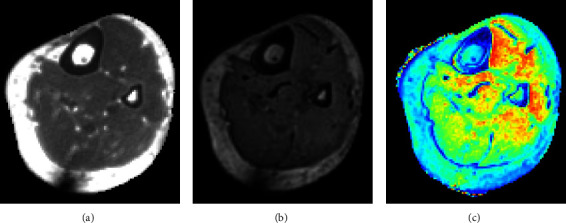
Example of an axial slice of a 3D dual-echo ultrashort echo time MRI scan of the tibia, similar to previously published methods. (a, b) correspond to the first and second echoes (TE_1_ = 50 *μ*s, TE_2_ = 4600 *μ*s), respectively. It is clear that the first echo has substantially more signal in the cortical bone region than in the second echo, whereas the signal in the soft tissue region has more subtle differences. (c) Depicts the porosity index, which is computed as the ratio between the echoes: PI (%) = TE_2_/TE_1_ *∗* 100. Although it is not obvious in the first or second echo, the PI clearly highlights the spatial variations of bone porosity in the tibial cortex [94].

Another clinically viable UTE-derived biomarker for assessing cortical bone health is called the Porosity Index (PI). The PI is defined as the ratio in the cortical region between two different echoes, one long and one short. Since the short echo contains signal from both water that is tightly bound to collagen and water that is freely moving in pores while the long echo only contains signal from the freely moving pore water, this produces an effective measurement of cortical porosity (Figure 8) [95]. The PI is known to strongly correlate with actual volumetric bone quality. This technique was validated in a study comparing the porosity index of the mid-tibia cortical bone samples of 16 donors with porosity ascertained using ground-truth micro-CT imaging. The investigators noted a strong correlation between the PI and the micro-CT imaging porosity, as well as strong reproducibility [10]. This demonstrated that it was feasible to use porosity indices to assess changes in cortical bone porosity in response to disease or therapy. UTE measurements of bone water and cortical porosity have demonstrated considerable value in the clinical assessment of bone and would likely also provide useful information for sports medicine.

UTE MRI is being utilized in the sports medicine clinic currently to monitor postoperative healing and evaluate tendinopathy. Chu and colleagues used UTE mapping to demonstrate changes in deep cartilage tissue health two years after anatomic ACL reconstruction, finding that about 3 of 4 ACL-reconstructed patients had intact central medial femoral condyle cartilage, and that the UTE values of the cartilage varied significantly with injury status and arthroscopic grade. The authors were optimistic that these techniques could be used to identify joints at risk for rapid regeneration and subsequent osteoarthritis [96]. A follow-up study showed that UTE MRI could be used to show progressive maturation of the ACL graft 2 years following anatomic reconstruction [97]. Another group sought to compare the results of anatomical lateral ankle ligament reconstruction with tendon allograft versus autograft with clinical scoring criteria as well as UTE MRI. They recruited 26 patients for this study and found that UTE showed higher values for the allograft group, suggesting to the authors that the autograft group had better outcomes in terms of revascularization, collagen structure, water contents, and tendon properties [98].

In addition to postoperative evaluation, UTE has proven useful in evaluating various tendinopathies. One group of investigators asked if UTE MRI could be used to distinguish between healthy and diseased Achilles tendons. They recruited ten patients with symptomatic Achilles tendons as well as ten healthy controls and scanned their Achilles tendons in four different locations with UTE MRI. The patients were also assessed with clinical metrics such as the Achilles Tendon Rupture Score (ATRS) and the American Orthopedic Foot and Ankle Society (AOFAS) score. They found that the UTE-T2 scores significantly differed between the pathologic Achilles tendon and the healthy Achilles tendon. They also found a rough correlation between the UTE-T2 scores and the traditional clinical metrics, suggesting to the authors that UTE-T2 scores might be a promising marker to detect and diagnose Achilles tendinopathy [99]. Patellar tendinopathy has also been looked at using UTE MRI. A population of 65 athletes with patellar tendinopathy was evaluated with UTE MRI to determine if it was feasible to get quantifiable T2 scores with a method that had adequate test-retest reliability. They found that quantitative multicompartment T2 analysis in patellar tendinopathy can be done using a voxel selection method that is based on biexponential fitting parameters [100]. Studies like this are critical as they establish a protocol that can be used by researchers in the field moving forward. There is a clinical trial underway currently that aims to evaluate whether autologous bone marrow expanded mesenchymal stem cells implanted into a pathologic patellar tendon will restore function as compared to a control group implanting autologous leukocyte-poor platelet-rich plasma. The investigators plan to evaluate this using UTE MRI [101]. Although certainly cost, scanning time, and technical challenges remain for UTE MRI, these studies demonstrate its value in clinical and research settings, as well as foreshadow future applications of this exciting novel technology.

## 10. Perspective

Several novel imaging modalities have been presented, providing further insight into managing bone injuries with new methods of monitoring healing and fracture risk. Awareness of these technologies and their utility has enabled an innovative transition from the research and clinical settings to early adoption across team sports at elite levels. In the professional sports medicine environment, imaging is often readily available and can serve as the basis for many critical decisions pertaining to an athlete's current health, risk, return to play, and even career longevity. Conventional radiographs, CT, MRI, and US all serve a role in the effective management of stress injuries to bone and their individual roles will undoubtedly increase in the coming decades, especially at the highest level of competition.

Point-of-care US is a cheap and effective technology that can be immediately performed on-site at a sporting event or training facility following an injury. This allows for early, accurate diagnosis of numerous pathologies along with monitoring of healing through serial imaging [57, 102], and it has shown excellent results in early fracture diagnosis over X-ray or CT [54, 57]. In particular, the US can identify callus development in stress injuries before radiographic changes are evident. US is also a reliable tool in injury tracking and can monitor all phases of fracture healing and identify potential problems, which is critical in the fast-paced, high-pressure landscape of elite sports. Concerning findings identified on point-of-care US in correlation with clinical examination can also warrant further advanced imaging evaluation, such as MRI, for information on the extent of bone involvement. Utilization of color and power Doppler functionality on US also provides useful information on early vascularization as a part of the bone healing evolution [54]. Diagnostic ultrasound allows for informed decisions on rehabilitation progression and supports return-to-play decisions or potential restrictions. While many professional teams and athletic centers have already adopted point-of-care US, its application will, in all likelihood, continue to grow over the coming years, and it may become standard for all top competitive teams.

The application of CT- or MRI-based bone quantification and FEA in sports medicine is also projected to increase over the coming years. High-profile athletes represent crucial assets to professional organizations wherein medical information gets immediately relayed to all stakeholders, including the physician, sports medicine and performance staff, coaches, front office members, and agents, in addition to a consulting physician if a second opinion is required. For instance, the “dreaded black line,” the hallmark sign on X-rays for anterior tibial cortex stress fractures, was classically seen as fractures in the bone. More recently, these fracture lines were examined microscopically in athletes indicating the fissures were in fact low-density bone undergoing intense remodeling, meaning healing was already underway, but the region was mechanically insufficient to handle normal stress [103]. Following any stress fracture, the primary questions are how the healing progresses and when an athlete can safely return to training or play. Delayed healing pertaining to bone injuries can raise discussions around operative or nonoperative management. While these decisions are often made in a subjective manner, CT- or MRI-based quantitative assessment of bone could provide the needed objectivity to bring these out of the research and clinical setting and into the forefront of bone injury management in elite sports.

The primary application of MDCT and HR-pQCT in sports medicine is the imaging and quantifying bone microstructure at distal sites and the extremities. This includes the tibia, and distal radius, as well as all the bones of the foot including the metatarsals. As mentioned previously, bone microarchitectural images enable representative quantifications which are related to disease state, fracture history, and risk, as well as nutritional health. Since MDCT scanners are widely available and HR-pQCT scanners are becoming increasingly more prevalent, these methods can be readily adopted to all competition levels to track healing progress longitudinally, as well as evaluate the impact of different training regimens and dietary changes on fracture risk. High-resolution MRI scans enable imaging of trabecular bone that is analogous to the quality of CT images without subjecting the patient to ionizing radiation. MRI can also image trabecular microstructure in deeper tissues like the proximal femur which are unattainable via CT due to the radiosensitivity of the pelvis. Cortical bone composition and mechanical parameters can be quantified with UTE MRI. However, MRI scans are more expensive and take longer than CT scans, and specialized high-resolution or UTE bone sequences are somewhat limited, so it is likely that MRI methods will be more applicable in research and in clinical practice at the professional level. As the state-of-the-art method for assessing bone strength, image-derived FEA can provide impactful quantitative and qualitative information to practitioners by generating bone mechanical parameter estimations, as well as 3D strain maps which indicate areas of structural vulnerability under loading. The data can provide an evaluation of the percent change in bone strength after each serial scan and is particularly useful in tracking bone healing if a healthy baseline scan is available for comparison. These metrics provide objective evidence of bone healing as a means to aid practitioners in making more informed decisions on whether to continue to advance rehabilitation or delay intense activity. Although CT- or MRI-derived FEA provides the most accurate methods available for quantifying bone health, these methods are expensive and require specialized software, extensive user expertise, and computation time. Moreover, further research is necessary to identify the optimal bone healing threshold sufficient to return to rigorous activity. As such, initial adoption of these methods will ostensibly come from professional teams with elite athletes whose health is inextricably linked with their team's financial future. Nevertheless, it is anticipated that these methods will see rapid expansion in the field of sports medicine as a whole in the coming decades.

## 11. Conclusion

The current standard practice for the evaluation of bone injuries in sports medicine relies primarily on qualitative assessments. The imaging methods outlined above have the ability to provide quantitative information to improve decisions and, ultimately, outcomes in the sports medicine field. As always, it is important to note that routine imaging can have its challenges around providing objective markers for healing, especially with slower-healing bone stress injuries. It is conceivable in these situations that quantitative imaging data could be supplemented with other blood-based biomarkers and patient feedback to further improve outcomes. In addition, further research is needed to delineate normative quantitative bone data for each modality in the sports populations. While normative data exist for DXA in osteoporotic subjects, there is no anthropometric baseline data for athletic cohorts, and further research is needed to fill this void before informed decisions can be made. Furthermore, radiology technicians consistently attempt to provide reliable images, but human error can lead to issues emulating the exact image registration, motion artifacts, parameters, and exposure. Moreover, imaging parameters such as resolution, scanner model, and sequence can impact quantitative measures, so uniform and reproducible protocols must be enhanced to enable comparison between different imaging sites.

## Figures and Tables

**Table 1:** Summary of the bone imaging modalities for the distal radius or tibia and deeper osseous structures such as the femur and vertebrae.

Modality	Extremities-distal radius and tibia					Deep structures-femur and vertebrae				
	DXA	HR-pQCT	MDCT	US	MRI	DXA	HR-pQCT	MDCT	US	MRI
Ionizing radiation (*μ*Sv)	<10	<5	10	0	0	<10	None	<3000	0	0
Resolution (*μ*m)	1000	80	5500	150	150	1000	None	1000	200	400
3-Dimensional	No	Yes	Yes	Yes	Yes	No	None	Yes	Yes	Yes
Scan region (cm)	10	10	10	4	1.5	10	None	20	4	10
Scan time (min)	10	3	<1	Varies	5	10	None	<1	Varies	10
Availability	Widely available	Limited	Widely available	Widely available	Widely available	Ubiquitous	None	Widely available	Widely available	Widely available
